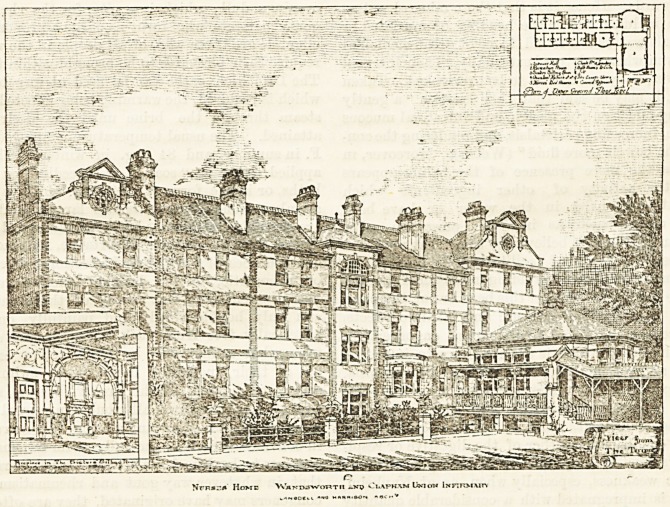# Hospital Construction

**Published:** 1899-10-07

**Authors:** 


### The Institutional Workshop.

### HOSPITAL CONSTRUCTION.

### NURSES' HOME AT THE WANDSWORTH AND CLAPHAM UNION INFIRMARY.

One of the most impressive points connected with our present-day hospital construction and management is the superior way in which the nurses are cared for in the matters of accommodation, board, leave of absence, and amusement. The London union infirmaries have felt the upward tendency, and are, in some instances at least, no whit behind the metropolitan general hospitals.

The most recent annexe for nurses is now being built at the Wandsworth and Clapham Infirmary at St. John's Hill, and we give with this description the ground and first floor plans of it. It is joined to the main building by a corridor, which in its turn is connected with the principal corridor of the wards, and it can also be approached from the terrace at the northern end of the infirmary. The corridor of communication leads directly into the vestibule and entrance hall of the Home, and on the right, on entering, are a large recreation-room and a sitting-room for the sisters. Another corridor divides the ground floor of the Home into nearly equal parts longitudinally, and the nurses rooms are ranged on either side. The main staircase is near the centre to the front, and the bath-rooms and closets are placed nearly opposite and to the back. There are two fire-escape staircases, and one adjoins these bath-rooms, while the other is at the extreme end of the block. There are 13 nurses' rooms on this floor, and also a large sitting-room for the assistant matron.

The first floor is very similar to the ground floor; but the space over the sister's sitting-room and part of the space over the recreation-room have been utilised for more rooms, there being 22 on this floor.

The second floor again is similar to the first, and the third contains the night nurses' rooms. These have been carefully shut off from the main staircase, so that they may be as quiet as possible. Each nurse has a minimum of 100 feet of floor space, and each room has an open fireplace, a fresh air inlet, and an extraction ventilator. The total accommodation is for 75 nurses.

The building is "constructed upon a fire-resisting principle," but we are not told what that principle is. The recreation-room and the corridors will be warmed by hot-water radiators as well as by open fireplaces. The architects are Messrs. Lansdell and Harrison, and the contractors Messrs. Gregory and Co. The foundation-stone of the building was laid on November 6th last year by Mrs. Breward Neal, wife of the able medical superintendent of the infirmary.

Nurses' Home Wandsworth and Clapham Union Infirmary

## Figures and Tables

**Figure f1:**
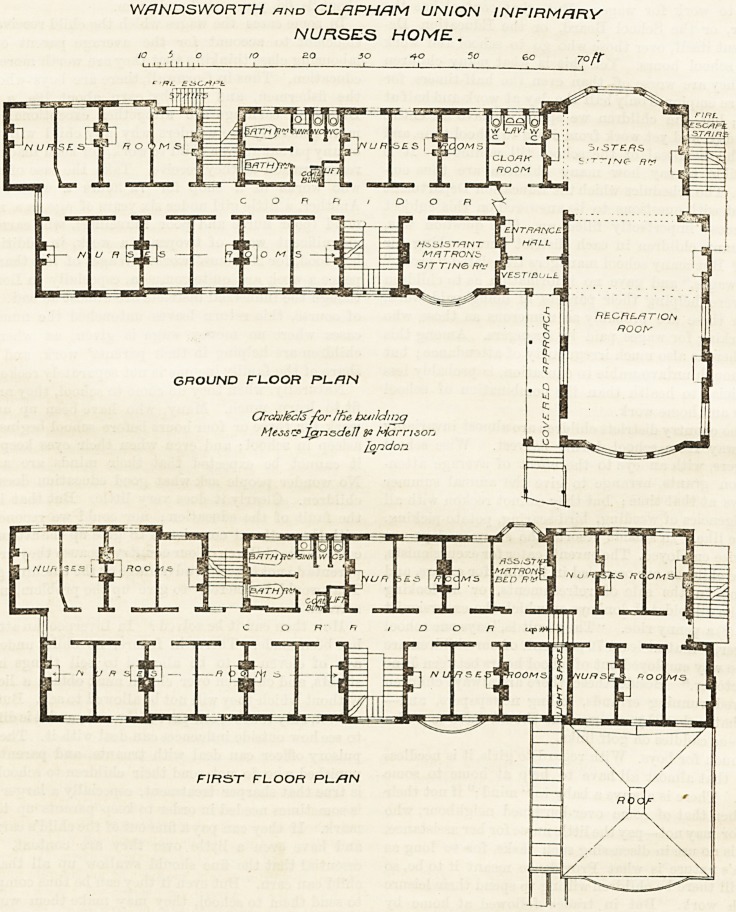


**Figure f2:**